# The impact of spinal anaesthesia on perioperative opioid consumption, postoperative pain and oncological outcome in radical retropubic prostatectomy—a retrospective before-and-after effectiveness study

**DOI:** 10.1186/s13741-022-00281-0

**Published:** 2022-10-03

**Authors:** Sandra Funcke, Xenia Schick-Bengardt, Hans O. Pinnschmidt, Burkhard Beyer, Marlene Fischer, Ursula Kahl, Rainer Nitzschke

**Affiliations:** 1 Department of Anesthesiology, Center of Anesthesiology and Intensive Care Medicine, University Medical Center Hamburg-Eppendorf, Martinistr. 52, 20246 Hamburg, Germany; 2 Institute of Medical Biometry and Epidemiology, University Medical Center Hamburg-Eppendorf, Hamburg, Germany; 3 Martini-Klinik, Prostate Cancer Center, University Medical Center Hamburg-Eppendorf, Martinistrasse 52, 20246 Hamburg, Germany

**Keywords:** Prostatectomy, Spinal anaesthesia, Opioid effect, Postoperative Pain, Biochemical cancer-free survival

## Abstract

**Background:**

Spinal anaesthesia preceding general anaesthesia has been conducted for open radical retropubic prostatectomy (RRP) to decrease immediate postoperative pain for many years. Nevertheless, the effectiveness of spinal anaesthesia to reduce postoperative opioid requirements remains unknown. The aim of the present study was to determine the effect of spinal anaesthesia preceding general anaesthesia on opioid requirements, postoperative pain and biochemical cancer-free survival.

**Methods:**

This before-and-after effectiveness study investigated effects of two different anaesthesia techniques in 636 patients with RRP. Three hundred eighteen consecutive patients in the SPA group (spinal anaesthesia preceding general anaesthesia) were compared with 318 patients in the GA group (general anaesthesia alone). The primary endpoint of the study was opioid consumption in the post-anaesthesia care unit. Secondary endpoints were intraoperative opioid consumption, postoperative pain, postoperative recovery time, the length of hospital-stay, persistence of pain 1 year after surgery and cancer-free survival. Differences between the groups were analysed by a two-sided t-test, *χ*^2^-test, Fisher’s exact test and Mann–Whitney *U* test and the influence of possible confounders on opioid consumption with a general linear model. Cancer-free survival was determined by Kaplan–Meier curves and group differences by log-rank tests and multivariable Cox regression analyses.

**Results:**

The total amount of morphine equivalent administered postoperatively was 7.5 [6.9; 8.1] mg in the SPA group and 6.0 [5.5; 6.5] mg in the GA group (mean [95% CI], *p* < 0.001). The amount of intraoperative sufentanil was 56.9 [55.1; 58.7] μg in the SPA group and 84.5 [82.5; 86.5] μg in the GA group (mean [95% CI], *p* < 0.001). There was no difference found in the postoperative pain level, length of hospital-stay and pain level 1 year after surgery. Biochemical cancer-free survival was highly related to TNM stage (*p* < 0.001, pT3 vs. pT2 hazard ratio 5.4 [95%CI 3.3; 9.2]) but not to the type of anaesthesia (*p* = 0.29).

**Conclusions:**

Spinal anaesthesia preceding general anaesthesia for RRP is associated with increased postoperative opioid consumption compared to general anaesthesia alone. Postoperative pain level and the oncological outcome are not affected by the adjunctive use of spinal anaesthesia. Thus, the addition of spinal anaesthesia to general anaesthesia has no advantage in RRP.

**Trial registration:**

ClinicalTrial.gov, NCT03565705.

## Introduction

Open retropubic radical prostatectomy (RRP) for localized prostate cancer has been performed with different anaesthesia techniques in the last 70 years. Soon after Millin first described RRP in 1945, in the 1950s, the procedure was performed under neuraxial anaesthesia alone in many surgical departments (Albertsen 2005; Morris and Candy 1957; Gardner 1958; Joshi et al. 2015). Today, general anaesthesia is regularly chosen for RRP and is often combined with neuraxial anaesthesia. The potential impact of neuraxial regional anaesthesia on major abdominal oncologic surgery is a reduction of intraoperative bleeding, reduced neuroendocrine stress response to the surgical trauma, less opioid requirements, less immunosuppression, reduced postoperative pain and cancer recurrence. (Shir et al. 1994; Brown et al. 2004; Salonia et al. 2006; Dale 1953; Kofler et al. 2019). This has been proposed against the background that major oncological surgery induces a neuroendocrine, metabolic and cytokine response, resulting in an immunosuppression in the perioperative period. (Kim 2017). Additional immunosuppressive effects are proposed for intraoperatively and postoperatively administered opioids (Kim 2017; Gottschalk et al. 2010; Beilin et al. 1996).

With increasing awareness of the side effects of opioids, the need for reducing opioid use has become more important in recent years (Hayhurst and Durieux 2016; Brown et al. 2018). For enhanced recovery after surgery (ERAS), it is recommended to minimize the use of postoperative opioids to a minimum. Neuraxial anaesthesia might reduce the neuroendocrine stress response to the surgical trauma and reduce the intraoperative and postoperative use of opioids (Gottschalk 2010; Snyder 2010). Nevertheless, adding a second anaesthetic regimen leads to additional risk factors, and this must be weighed against the benefits. Previous studies produced controversial results regarding the benefit of additional neuraxial anaesthesia on tumour recurrence rates in RRP (Lee et al. 2015; Weng et al. 2016; Le-Wendling et al. 2016; Wall et al. 2019). Most of these studies investigated epidural anaesthesia and only a minor number of studies concentrated on spinal anaesthesia (Tseng et al., 2014; Ehdaie et al., 2014; Roiss et al., 2014). Besides, recently published studies emphasized the role of the amount of intravenously infused opioids (Yardeni et al. 2008; Zylla et al. 2013; Kim, 2017). And although the intraoperative amount of opioid had been reported regularly, the intraoperative opioid dose was usually not related to postoperative opioid consumption.

The aim of the present before-and-after effectiveness study was to assess postoperative and intraoperative opioid consumption, postoperative pain, recovery time, the length of hospital-stay, persistence of pain 1 year after surgery and cancer-free survival after open RRP in a cohort of patients with a combination of spinal anaesthesia and general anaesthesia compared to a cohort with only general anaesthesia. We hypothesized that patients with a spinal anaesthesia preceding general anaesthesia had less morphine equivalent administered in the post-anaesthesia care unit and had a longer biochemical cancer-free survival than patients with general anaesthesia alone.

## Methods

### Study design and setting

Within the prospectively collected, institutional review board-approved database, the authors included 632 consecutive patients who underwent elective open RRP in the Martini-Klinik Prostate Cancer Center affiliated with the University Medical Centre Hamburg-Eppendorf. The study was approved by the local ethics committee of the Medical Board Hamburg (WF-040/17, July 11, 2017), and the trial was registered at ClinicalTrial.gov (Identifier ‘NCT03565705’). All patients gave consent to participate in the prospectively collected database. The manuscript adheres to the applicable SQUIRE 2.0 (Revised Standards for Quality Improvement Reporting Excellence) guidelines from September 15, 2015.

With more than 2500 RRPs carried out annually, the Martini-Klinik is in terms of the number of operative procedures the world’s largest prostate cancer clinic. Twelve specialized surgeons perform RRP either by open or robotically assisted minimally invasive procedures. Before March 1, 2017, the standard anaesthesia technique for open RRP in the Martini-Klinik consisted of spinal anaesthesia combined with general anaesthesia. That standard anaesthesia technique was changed on March 1, 2017, from the combination of general anaesthesia preceded by spinal anaesthesia to general anaesthesia alone. Data were collected from the last 318 consecutive men who underwent open RRP before March 1, 2017, with spinal anaesthesia (SPA), and the cohort of the next 318 patients after March 1, 2017, under general anaesthesia only (GA).

### Patient population

We included only male patients aged 18 years or older, because the study investigated opioid consumption during and after open radical retropubic prostatectomy. Patients with chronic pain therapy (e.g., out-of-hospital opioid therapy) were excluded from the study. The authors included all patients who received only general anaesthesia without spinal anaesthesia after March 1, 2017, and all patients who received a combination of both until February 28, 2017.

### Anaesthesia management

All patients with and without intraoperative spinal anaesthesia received the same perioperative care following local standards. Upon arrival in the operation room, the anaesthesiologists started convective air-warming to minimize perioperative hypothermia. Routine monitoring included electrocardiography, non-invasive blood pressure measurement, pulse oximetry, Bispectral index monitoring (BIS), acceleromyography for train-of-four (TOF) counts, body temperature measurement (a probe placed in the oesophagus in the GA group, and above the laryngeal mask in the SPA group) and capnography in both groups.

The patients in the SPA group received spinal anaesthesia before induction of general anaesthesia. The sterile lumbar puncture was carried out on the awake patient sitting on the operating room table using a standard 26-gauge pencilpoint needle between L2 and L3 or between L3 and L4 after local anaesthesia on the puncture site. Spinal anaesthesia was achieved by injecting isobaric bupivacaine 0.5% with a volume between 2.8 and 3.5 ml (14 to 17.5 mg, depending on the patient’s height) in the cerebrospinal fluid. The placement and pharmacological effect were confirmed by the loss of motor function in the lower extremities. Dermatomal levels were determined by testing the patient’s ability to discriminate between heat and cold. After clear confirmation of the presence of spinal anaesthesia, the patients in the SPA group received a sufentanil bolus of 0.5 μg·kg^−1^ followed by a propofol bolus of 2 mg·kg^−1^ body weight. The airway was secured by inserting a third-generation laryngeal mask.

For the induction of general anaesthesia, the patients in the GA group received a sufentanil bolus of 0.5 μg·kg^−1^ followed by a propofol bolus of 2 mg·kg^−1^ body weight and rocuronium 0.5 mg·kg^−1^. The airway was secured with tracheal intubation.

After airway management positive pressure ventilation was started, and anaesthesia maintenance was performed with either sevoflurane 1.7–2.0 Vol% (minimal alveolar concentration MAC 1.0) or propofol infusion both with a target range of BIS (bispectral index, depth of sedation monitoring) values between 40 and 50. Further application of sufentanil was administered at the discretion of the attending anaesthesiologist using clinical signs of nociception (e.g., increase in heart rate or blood pressure). Patients received continuous low-dose norepinephrine administration compensating for the vasodilatation associated with narcosis to maintain the mean arterial pressure above 65 mmHg. At the end of surgery, patients in both groups received 1 g metamizole dipyrone i.v. and were transferred from the operation room into the post-anaesthesia care unit (PACU) after extubation.

During the postoperative period in the PACU, patients in both groups received postoperative care following the institution’s standardized treatment protocol. Upon arrival at the PACU, the patient’s level of analgesia was measured on a numerical pain rating scale (NRS) from 0 to 10. NRS ≤ 3 was considered to be no or mild pain, and NRS > 3 was considered to be moderate to intense pain (Hayhurst and Durieux 2016). The NRS was reassessed every 15 min. In cases of NRS > 3 or whenever the patients mentioned pain, patients received a bolus of 3.75 mg piritramide (dose equivalence to morphine is 1:0.7 according to 2.625 mg morphine) with no upper dose limit. On the ward, all patients from both groups received the same, regular and precisely scheduled, prophylactic analgesic treatment plan during the first 2 days after surgery. This multimodal therapy included scheduled, weight-adjusted non-opioid analgesics, anti-spasmic medication and mobilizing physiotherapy.

### Outcome measures

The primary endpoint of the study was the total amount of postoperative opioid consumption (morphine equivalents) in the PACU. Secondary endpoints were the extent of intraoperative opioid consumption (sufentanil during surgery), maximum postoperative pain measured with the highest score on the NRS, duration of postoperative recovery time (time interval between postoperative tracheal extubation and the patient fulfilling the fit-for-discharge criteria from the PACU to the ward), days in hospital and the occurrence of PONV or shivering in the PACU. Long-term parameters were cancer-free survival and persistence of pain 1 year after surgery. In addition, we collected further perioperative parameters (e.g., blood loss, need for vasoactive agents) to assess the comparability of the two treatment groups.

### Sample size calculation

The sample size calculation was based on a chart review of routine data of the patients having had a prostatectomy between February and March 2017 in the author’s institution due to a lack of adequate published data. In this sample, the authors found a mean consumption of morphine equivalence in the PACU in patients with spinal anaesthesia of 7.7 mg and considered a difference of 20% (i.e., 1.5 mg) to be of clinical relevance. Based on the standard deviation of 5.5 mg in the random sample, the authors calculated that a total sample size of 636 patients (318 per group) has a 90% power to detect differences between the group means at a 5% significance level in a two-sample *t*-test using the software package PASS 2008 version 08.0.6 (NCSS LLC, Kaysville, UT, USA).

### Statistical analysis

Differences in the primary endpoint were assessed by a two-sided *t*-test. Additionally, to adjust for potential confounders and group imbalances, the influence of the study group and 17 covariates as fixed effects on the postoperative opioid consumption in the PACU were evaluated with a general linear model. The model was adjusted for 17 baseline variables: age, BMI, ASA status and each patient’s preoperative long-term medication (in 14 categories). Data distributions were assessed graphically via histograms. Analysis of the demographic and procedural data was performed using Mann–Whitney *U* test or Fisher's exact test as appropriate. The secondary endpoints were analysed in an explorative manner by two-sided t-tests or Mann–Whitney *U* tests for continuous data or by *χ*^2^-tests for categorical data. Cancer-free survival was determined by Kaplan–Meier curves and group differences by log-rank tests. Effects of additional covariates were examined by multivariable Cox regression analyses. Statistical analyses were performed using SPSS 25.0 (IBM SPSS Statistics Inc., Armonk, NY, USA), and two-tailed *p*-values < 0.05 were considered significant for the primary endpoint. Secondary endpoints were compared in an explorative analysis.

## Results

To analyse the postoperative opioid consumption from 636 patients, the authors included the last 318 patients before March 1, 2017 (September 20, 2016, until February 28, 2017), who received spinal anaesthesia preceding general anaesthesia. The 318 consecutive patients who received general anaesthesia without spinal anaesthesia were included from March 1, 2017, until June 22, 2017. Patient characteristics of the study population are provided in Table 1 and intraoperative data in Table 2.

**Table 1:** Baseline characteristics

	**SPA** (*n* = 318)	**GA** (*n* = 318)	***P*-value**
Age [years]	64 ± 7.0	64 ± 7.3	0.093
Height [cm]	179 ± 6.0	179 ± 6.7	0.408
Weight [kg]	86 ± 10.6	87 ± 11.7	0.130
BMI [kg·m^−2^]	26.7 ± 3.0	27.0 ± 3.1	0.379
Hb before surgery [g·dl^−1^]	14.7 ± 1.0	14.7 ± 1.0	0.921
**ASA physical status class**			< 0.001
II	268 (84.3)	221 (69.5)	
III	50 (15.7)	97 (30.5)	
**Preoperative medication**			
Betablocker	40 (12.6)	67 (21.1)	0.006
Diuretics	21 (6.6)	41 (12.9)	0.011
Antiarrhythmics	2 (0.6)	7 (2.2)	0.177
Metabolic	81 (25.5)	107 (33.6)	0.03
ASS/anticoagulants	16 (5.0)	72 (22.6)	< 0.001
Analgesics	6 (1.9)	8 (2.5)	0.788
Antihypertensives	114 (35.8)	148 (46.5)	0.008
Urological	51 (16.0)	46 (14.5)	0.659
Pulmonary	16 (5.0)	14 (4.4)	0.852
Neurological	15 (4.7)	14 (4.4)	1.0
Gastroenterological	35 (11.0)	39 (12.3)	0.711
Ophthalmological	8 (2.5)	12 (3.8)	0.497
Others	24 (7.5)	52 (16.4)	0.001
None	104 (32.7)	62 (19.5)	< 0.001

Values are mean ± SD for continuous data or n (%) for categorical data. *P*-values are displayed as results from nonparametric analysis (by Mann–Whitney *U* test) for metric variables and from Fisher’s exact test for categorical data (ASA class and preoperative medication)

*SPA group* Spinal anaesthesia preceding general anaesthesia, *GA group* General anaesthesia alone, *BMI* Body mass index, *Hb* Haemoglobin level, *ASA* American Society of Anesthesiologists

**Table 2:** Intraoperative data

	**SPA** (*n* = 318)	**GA** (*n* = 318)	***P*-value**
Duration of surgery [min]	180 [160–205]	175 [150–205]	0.058
Induction bolus of propofol [mg]	200 [160–200]	200 [70–200]	0.962
Depth of sedation [BIS]	39 [35–43]	40 [37–43]	0.13
Blood loss [ml]	800 [600–1200]	800 [600–1000]	0.032
Minimum Hb level [g·dl^−1^]	11.1 [9.1–12.1]	11.9 [10.6–12.5]	0.17
Amount of crystalloid fluid [ml]	4500 [4000–5000]	4500 [4000–5000]	0.507
Amount of colloid fluid [ml]	0 [0–500]	0 [0–500]	0.185
Body temperature [°C]	35.9 [35.5–36.1]	36.0 [35.6–36.3]	< 0.001
Number of pain ratings with NRS > 3	1.0 [0–2]	0.5 [0–1]	0.348
Hb after 24 h [g·dl^−1^]	11.4 [10.5–12.1]	11.4 [10.6–12.1]	0.751

Values are median [Interquartile Range]. *P*-values are displayed as results from group comparison by Mann–Whitney *U* test

*SPA group* Spinal anaesthesia preceding general anaesthesia, *GA group* General anaesthesia alone, *BIS* Bispectral index, *Hb* Haemoglobin level, *NRS* Numeric Rating Scale

**Fig. 1:**
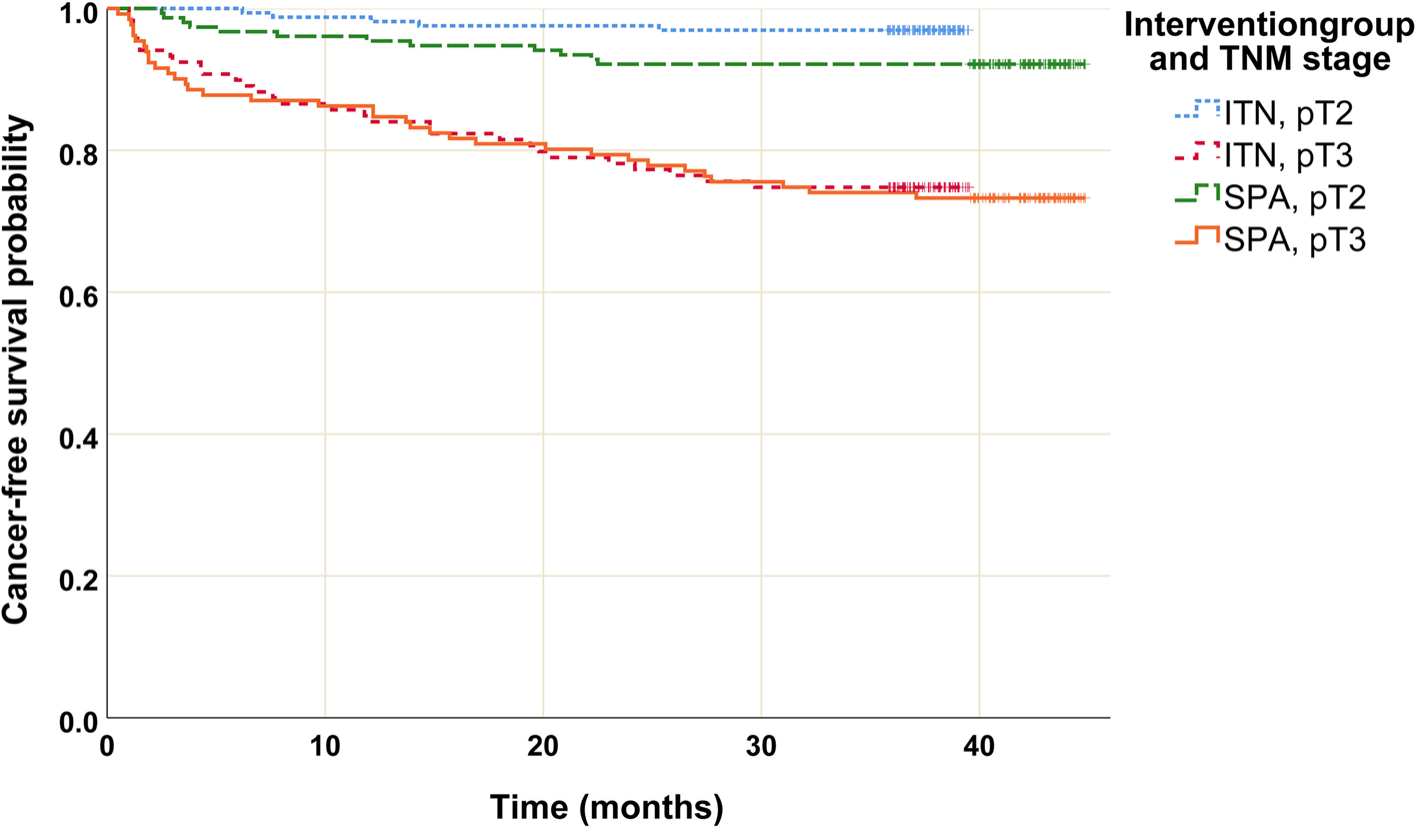
Kaplan–Meier curves of cancer-free survival after radical retropubic prostatectomy. Kaplan–Meier curves of cancer-free survival by study group and tumour stage (TNM classification, pT2: within the prostatic capsule, pT3 extracapsular extension) over time. In multivariable Cox regression analyses, the time to cancer recurrence was highly associated with TNM stage (*p* < 0.001, pT3 vs. pT2 hazard ratio 5.4 [95%CI 3.3; 9.2]), but not with group (*p* = 0.29), age (*p* = 0.737), ASA class (*p* = 0.935) and BMI (*p* = 0.779), and thus those four variables were excluded from the final model. Pairwise group comparisons between tumour stages were conducted by Kaplan–Meier analysis with log-rank test: pT2a vs. pT2c *p* = 0.211, pT2a vs. pT3a *p* = 0.06, pT2a vs. pT3b *p* = 0.01, pT2c vs. pT3a *p* < 0.01, pT2c vs. pT3b *p* < 0.01, pT3a vs. pT3b *p* < 0.01

The total amount of post-operative opioid, which was the primary endpoint of the study, differed between the two groups with the SPA group receiving on average 21% (95% CI: 10–32%) more morphine equivalents than the GA group (Table 3). A general linear model indicated that on average, the amount of morphine equivalents received was 1.3 (0.6–2.2) mg higher in the SPA group than in the GA group (mean (95% CI), *p* = 0.001) and was negatively related to age with − 0.11 [− 0.16 to -0.05] mg less morphine equivalents consumption per year increase in the patient’s age (mean (95% CI), *p* < 0.001). When choosing the study group as the only fixed effect neglecting the additional 17 confounders, the group effect was still comparable (1.5 (0.7; 2.22) mg (mean (95% CI]), *p* < 0.001). Regarding the covariates BMI, ASA status and the patient’s preoperative medications, no evidence of influence on post-operative opioid consumption was found. Table 3 further shows the secondary study endpoints. Due to slightly skewed data distributions, nonparametric testing by Kruskal–Wallis test was applied for global group comparisons of the variables in addition to the two-sample t-test. The results of the nonparametric testing were comparable. The data shows no evidence of a difference between the groups in the secondary endpoints. The biochemical recurrence-free survival varied depending on the pre-operative tumour state, but neither group nor age, BMI or ASA class had an influence (Fig. 1).

**Table 3:** Primary and secondary study endpoints

	**SPA** (*n* = 318)	**GA** (*n* = 318)	***P*-value**
Morphine equivalents in PACU [mg]	7.5 (6.9–8.1)	6.0 (5.5–6.5)	< 0.001*
Consumption of sufentanil [μg] during surgery	56.9 (55.1–58.7)	84.5 (82.5 -86.5)	< 0.001^†^
Maximum NRS in PACU	3.5 (3.2–3.7)	3.2 (3.0–3.5)	0.201^‡^
Postoperative recovery time [min]	166 (161–172)	163 (158–169)	0.467^§^
Occurrence of PONV	28 (12.6)	37 (17.5)	0.158
Occurrence of shivering	17 (8.4)	12 (7.2)	0.662

Values are mean (95% CI) for continuous data and *n* (%) for categorical data. *P*-values from group comparison by two-sided t-test for continuous data or *χ*^2^-test for categorical data. *P*-values from nonparametric testing (Mann–Whitney *U* test) in comparison: *P < 0.001, ^†^*P* < 0.001; ^‡^*P* = 0.126; ^§^*P* = 0.745

*SPA group* Spinal anaesthesia preceding general anaesthesia, *GA group* General anaesthesia alone, *PACU* Post-anaesthesia care unit, *NRS* Numeric Rating Scale, *PONV* Postoperative nausea and vomiting

## Discussion

This observational, before-and-after effectiveness study investigated a possible benefit of additional spinal anaesthesia added to general anaesthesia in open RRP. In the early recovery period, the SPA group received, on average, 21% more opioid in the PACU compared to the GA group. The SPA group received 23% less sufentanil than the GA group without spinal anaesthesia during the operation. No evidence was found for a difference between the patients with and without spinal anaesthesia regarding intraoperative blood loss, the postoperative pain level, duration of stay in the PACU, or occurrence of postoperative PONV and shivering. Furthermore, opioid consumption was dependent on the patient’s age with 1.1 mg less morphine equivalents per 10-year increase in the patient’s age. Addition of SPA to general anaesthesia had no influence on the long-term follow-up regarding persistence of pain 1 year after surgery and biochemical recurrence-free survival up to 3 years.

It is well known that the severity of pain both at rest and with activity correlates with a decrease in the patients’ quality of recovery in the immediate postoperative period (Bowyer et al. 2014). Regional anaesthesia techniques that have been used for improving analgesia and sparing opioids after RRP include epidural and spinal anaesthesia for more than half a century, and more recently, intrathecal opioid administration, transversus abdominis-plane block and local wound infiltration (Gardner 1958; Shir et al. 1994; Kofler et al. 2019; Fant et al. 2011; Andrieu et al. 2009; Yu et al. 2014; Tauzin-Fin et al. 2009). Some ERAS protocols suggest to use thoracic epidural anaesthesia for superior pain relief in open abdominal surgery (Cerantola et al. 2013). Although there are ERAS protocols for many major abdominal procedures, there is no specific protocol on RRP.

A systematic review of optimal pain management for RRP revealed a lack of evidence to develop an optimal pain management protocol and the need for more procedure-specific studies comparing the pain and analgesic requirements for surgical procedures (Joshi et al. 2015). Consequently, anaesthesiologists decide upon an anaesthesia technique using clinical considerations such as good conditions for the surgeon, minimal bleeding during the procedure and a fast postoperative recovery with the postoperative pain level as low as possible. In this context, it is of high importance to re-evaluate established treatment protocols regularly to contribute to finding optimized procedure-specific perioperative protocols.

Spinal anaesthesia has been preferred in several institutions because it provides good muscle relaxation, minimal bleeding during the procedure, a fast postoperative recovery and is reported to decrease postoperative pain and supplemental intravenous opioid demand compared to general anaesthesia (Kofler et al. 2019; Salonia et al. 2004). Brown et al. compared general anaesthesia with a general anaesthesia preceded by spinal anaesthesia with intrathecal administration of bupivacaine, clonidine and morphine (Brown et al. 2004). In their study, intrathecal analgesia decreased pain and postoperative intravenous opioid consumption, while haemoglobin on postoperative day 1 and blood transfusion as well as pain and functional status after discharge from the hospital did not differ between the groups. Likewise, other studies showed that spinal anaesthesia in combination with propofol sedation resulted in decreased intraoperative blood loss, a shorter PACU stay and lower pain scores in the PACU when compared to a general anaesthesia without regional anaesthesia (Salonia et al. 2006; Sved et al. 2005). A possible explanation would be that intrathecal opioids could have an additive pain reducing effect which has been proven for patients with cancer pain (Brogan et al. 2020). In contrast, our findings showed a higher need for postoperative intravenous opioid administration in the PACU in the SPA group, with comparable blood loss and pain scores. The omission of the intrathecal opioid injection in our patients might be an explanation for the differences between our results and previous studies. In some patients, the effect of spinal anaesthesia might already have diminished when patients arrived in the PACU, although bupivacaine is a long-acting local anaesthetic. Another possible explanation is that the difference in postoperative opioid consumption is due to the higher amount of intraoperative intravenous sufentanil in the GA group. In our patients, more high-potency intraoperative opioid administration was associated with less postoperative opioid consumption in the PACU. Therefore, the spinal anaesthesia preceding general anaesthesia in the SPA group might have led to a reduction in intraoperative opioid administration but to a higher need for postoperative opioid administration. In our data, there was no evidence for a difference between the study groups neither in the postoperative pain level in the early period after surgery nor 1 year after surgery.

The aim of opioid-sparing is not only to reduce side effects such as nausea, vomiting, obstipation and hypoventilation but also not to reduce the immune competence of patients after cancer surgery. There is reasonable evidence that high amounts of opioids might favour the neo-vascularization of a tumour and immunodeficiency by impaired cellular and humoral immune function in humans (Kim 2017; Ben-Eliyahu et al. 1999). Nevertheless, current study results on the long-term benefit of neuraxial regional anaesthesia on tumour progress after RRP are inconsistent (Lee et al. 2015; Weng et al. 2016; Wuethrich et al. 2010; Biki et al. 2008; Le-Wendling et al. 2016; Wall et al. 2019). On the one hand, some studies showed a trend towards increased overall and recurrence-free survival in the groups receiving neuraxial anaesthesia in addition to general anaesthesia after abdominal cancer surgery (Lee et al. 2015; Weng et al. 2016; Wuethrich et al. 2010). On the other hand, a meta-analysis on studies after prostatectomy and a recent narrative review claimed that the use of neuraxial anaesthesia was not associated with a longer biochemical recurrence-free survival (Ben-Eliyahu et al. 1999; Le-Wendling et al. 2016). In the present study, the multivariable Cox regression results indicated that the oncological outcome after radical prostatectomy was not affected by the adjunctive use of spinal anaesthesia and the pre-operative tumour status was the only variable related to the oncological outcome.

## Limitations

There are some limitations to our study. First, only male patients were included because of the choice to investigate patients undergoing prostatectomy. Second, patients in the SPA group were more likely to receive total intravenous anaesthesia with a laryngeal mask. Nevertheless, sedation monitoring revealed no differences in the depth of hypnosis between the groups. Next, the typical limitations of a retrospective chart review must be considered in this study design. The two cohorts in this study underwent an RRP at different time periods, and therefore, confounding of groups and time periods and bias by sequential enrolment cannot be ruled out. Further, a bias by selection must be carefully considered. Indeed, the study groups had different baseline characteristics. This was taken into account by adjusting for the above-mentioned variables in a general linear model. The model identified the variables ‘study group assignment’ and ‘patient’s age’ as the only variables with an effect on postoperative opioid consumption. Besides, the present study was not powered for the secondary endpoint biochemical recurrence-free survival. However, the relatively large population size and the prospectively collected long-term follow-up are strengths of this study.

## Conclusions

In conclusion, patients in the SPA group received more post-operative opioid in the PACU. Besides, preceding spinal anaesthesia before general anaesthesia for open RRP did not minimize blood loss, reduce postoperative pain levels or enhance short-term recovery. There were no differences in the long-term follow up with regard to pain persistence after one year or biochemical recurrence-free survival. Thus, the addition of spinal anaesthesia to general anaesthesia does not appear to be advantageous in RRP but increases the postoperative opioid consumption compared to general anaesthesia alone.

## Data Availability

The datasets used and analysed during the current study are available from the corresponding author on reasonable request.

## Abbreviations

ASA: American Society of Anesthesiologists
BIS: Bispectral index
BMI: Body mass index
ERAS: Enhanced recovery after surgery
GA: General anaesthesia
PACU: Post-anaesthesia care unit
PONV: Post-operative nausea and vomiting
RRP: Radical retropubic prostatectomy
SPA: Spinal anaesthesia
TNM: Tumour classification system
TOF: Train of four
